# Leveraging Genetic Reports and Electronic Health Records for the Prediction of Primary Cancers: Algorithm Development and Validation Study

**DOI:** 10.2196/23586

**Published:** 2021-05-25

**Authors:** Nansu Zong, Victoria Ngo, Daniel J Stone, Andrew Wen, Yiqing Zhao, Yue Yu, Sijia Liu, Ming Huang, Chen Wang, Guoqian Jiang

**Affiliations:** 1 Department of Health Sciences Research Mayo Clinic Rochester, MN United States; 2 University of California Davis Health Sacramento, CA United States

**Keywords:** genetic reports, electronic health records, predicting primary cancers, Fast Healthcare Interoperability Resources, FHIR, Resource Description Framework, RDF

## Abstract

**Background:**

Precision oncology has the potential to leverage clinical and genomic data in advancing disease prevention, diagnosis, and treatment. A key research area focuses on the early detection of primary cancers and potential prediction of cancers of unknown primary in order to facilitate optimal treatment decisions.

**Objective:**

This study presents a methodology to harmonize phenotypic and genetic data features to classify primary cancer types and predict cancers of unknown primaries.

**Methods:**

We extracted genetic data elements from oncology genetic reports of 1011 patients with cancer and their corresponding phenotypical data from Mayo Clinic’s electronic health records. We modeled both genetic and electronic health record data with HL7 Fast Healthcare Interoperability Resources. The semantic web Resource Description Framework was employed to generate the network-based data representation (ie, patient-phenotypic-genetic network). Based on the Resource Description Framework data graph, Node2vec graph-embedding algorithm was applied to generate features. Multiple machine learning and deep learning backbone models were compared for cancer prediction performance.

**Results:**

With 6 machine learning tasks designed in the experiment, we demonstrated the proposed method achieved favorable results in classifying primary cancer types (area under the receiver operating characteristic curve [AUROC] 96.56% for all 9 cancer predictions on average based on the cross-validation) and predicting unknown primaries (AUROC 80.77% for all 8 cancer predictions on average for real-patient validation). To demonstrate the interpretability, 17 phenotypic and genetic features that contributed the most to the prediction of each cancer were identified and validated based on a literature review.

**Conclusions:**

Accurate prediction of cancer types can be achieved with existing electronic health record data with satisfactory precision. The integration of genetic reports improves prediction, illustrating the translational values of incorporating genetic tests early at the diagnosis stage for patients with cancer.

## Introduction

Cancer is the second leading cause of death worldwide [1]. The health burden of cancer in the United States is substantial [2,3], with approximately 1.8 million new diagnoses and an estimated 600,000 deaths in 2020 alone [4]. Despite the advances in characterizing oncogenic mutations in the past few decades, overcoming the consequences of cellular self-renewal and neoplastic transformation remains a challenge in cancer therapy research [5]. Therefore, continued discoveries in causes, treatment, and management are needed to further the knowledge and understanding of this collection of related diseases [6].

Modern gene technology has provided an opportunity to identify certain gene mutations associated with increased cancer risk. Approximately 5% to 10% of all cancer diagnoses are linked to cancer predisposition syndromes [7-9]. Major syndromes of cancer disposition affecting adults include breast, ovarian, prostate, gastric, and pancreatic cancer [7]. Precision medicine initiatives call for the leveraging of clinical and genomic data to not only screen for cancers but also to help monitor cancer progression and guide therapy options [10]. Clinicians can facilitate early screening critical for risk assessment and surveillance [8]. If cancer is detected at an early stage, survival rates tend to be significantly higher than those for cancers diagnosed at an advanced stage [11-13]. Nash et al [11] cite figures as drastic as 90% survival for early ovarian cancer detection compared to only 5% survival with advanced stage detection, as an example. The utilization of genetic tests in diagnosing primary cancer also becomes critical when the symptoms and the physical exams suggest unspecified cancer known as cancer of unknown primary [14]. Cancer of unknown primary accounts for 3% to 5% of all tumors [15]. The prediction of the primary cancer of cancer of unknown primary can significantly increase our current knowledge of metastasis and benefit the treatment of patients with cancer of unknown primary.

The implementation and adoption of health information technology have given frontline clinicians access to a large repository of longitudinal clinical data collected during health care encounters [16,17]. Medical insight and clinical decision making rely heavily upon access to these data from electronic health records. Artificial intelligence techniques, such as machine learning methods, are promising for finding patterns and discovering associations in health care data to help predict diseases [18]. Improved predictions can be made by integrating diverse types of digital data in patients’ charts, which include diagnosis codes, clinical notes, laboratory test results, and treatment data [19].

As demand grows for genetic testing from patients and as genomic data continue to be incorporated into electronic health records, there is a need to study how genetic reports, along with electronic health record data, can be leveraged to predict cancers. Conventional computational methods for predictive models are based on features extracted from diverse data sources, known as bag of features [20]. The features in these models are treated independently, and the potential connections and patterns among the features cannot be fully explored to serve the prediction. A network-based data model can be used to represent the association between data models with edges, and the potential patterns are embedded in the topological structure of the network. Predictions from network-based data representations have achieved promising results in diverse biomedical areas, such as drug-target prediction [21] and patient clustering [22]. Representing correlations among phenotypic and genetic data elements through network-based data modeling shows great potential in cancer prediction.

The objective of this study was to harmonize phenotypic and genetic features for accurate and explainable cancer prediction, specifically: (1) developing a network-based framework with standard health care data exchange frameworks, the HL7 Fast Healthcare Interoperability Resources (FHIR) [23] and the Resource Description Framework (RDF) for graph-based data representations, (2) employing a state-of-the-art graph embedding algorithm, Node2vec [24], to obtain features for machine learning and deep learning models, and (3) implementing the proposed method with a collection of genetic reports of patients with cancer and the corresponding phenotypic data from Mayo Clinic’s electronic health record systems and comprehensive experiments.

## Methods

### Preliminary

FHIR is a standardized data framework designed for data exchange between different medical centers to enable information to be captured as it is generated, significantly simplifying population and real-time updates of predefined data models [23,25]. The FHIR specification defines a set of granular clinical concepts and resources to provide standard data infrastructure to support implementations [23]. FHIR-based data models are built upon combinations of these resources and a set of attributes with value types. The common attributes (eg, *identifier*) and unique attributes (eg, *bodySite*) in a resource are used to facilitate data modeling. Common data types (eg, *String* and *CodeableConcepts*) are used to constrain the attribute based on an adaptation of clinically related ontologies, such as SNOMED CT [26], LOINC [27], and International Statistical Classification of Diseases ninth (ICD-9) and tenth revisions (ICD-10) [28].

RDF is a general metadata or data model that defines concepts and web-resources based on a variety of syntax notations and data serialization formats [29]. Inherited from the classical conceptual modeling approaches, RDF utilizes the expressions to form triples, subject-predicate-object, to model data elements (eg, web resources). Specifically, in this study, the subject denotes the clinical data elements (eg, patients), and the predicate denotes a relationship between 2 data elements.

### Framework

**Figure 1:**
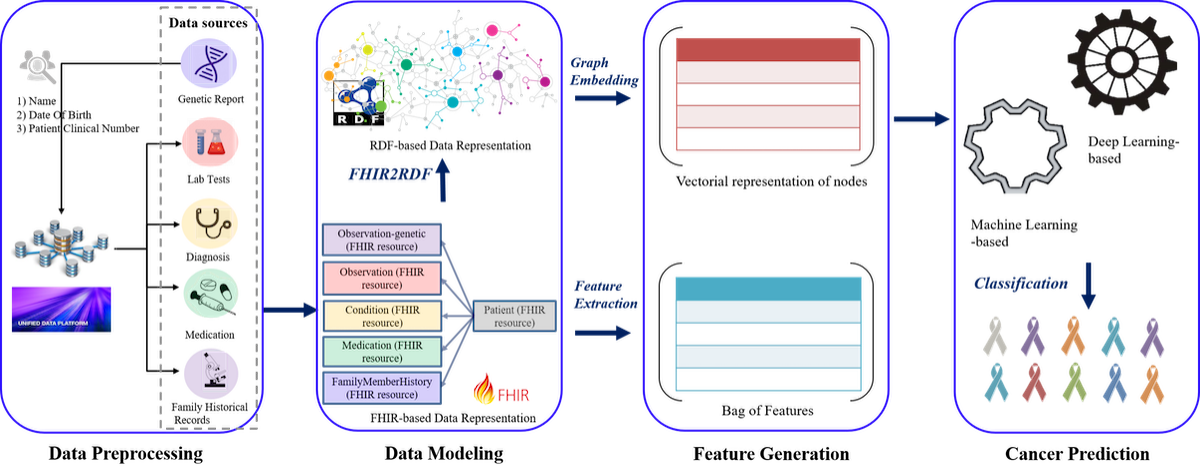
A network-based framework for cancer prediction based on Fast Healthcare Interoperability Resources and Resource Description Framework.

We proposed a network-based framework (Figure 1) that represented cancer data using the FHIR standard and RDF to facilitate the cancer prediction process. Five types of data sources extracted from the electronic health record—genetic information, lab tests, diagnosis, medication, and family historical records—were represented with FHIR resources and converted to the RDF-based representation. A graph-embedding algorithm, Node2vec, was used to provide a vectorial representation of nodes in the resulting network along with bag of features to form the features for the classification models.

### Data Preprocessing

Genetic data were extracted from 1011 aggregated anonymized genetic test results (Foundation Medicine Inc), including microsatellite instability and tumor mutational burden. Medical record data elements related to laboratory results, diagnoses, medications, and family histories were extracted from approximately 515,000 billing encounters (666,000 electronic health record encounters) retrieved from a Mayo Clinic clinical data warehouse of [30]. We integrated genetic and electronic health record data by mapping patient information based on 3 data elements: patient clinic number, names (first and last name), and date of birth. Lab tests, diagnosis, medication, and family historical records were searched based on the mapped patients. We used natural language processing to normalize the names and values. For *diagnosis* and *medication*, all diseases and medications were represented with standardized names encoded by ICD-9 [31] and RxNorm [32] codes. For lab tests, we represented all the tests with standard names encoded by LOINC [27]. For *family historical records*, each record was processed by a pipeline (NLP2FHIR [33]), where the medical concepts were identified and normalized using cTAKES [34], MedXN [35], and MedTime [36]. We encoded the diseases from family historical records using ICD-9 codes. To build the data set utilized for the cancer prediction, all the records within the billing circle related to the target cancers were removed. The top 10 elements in each data source can be found in Table 1.

**Table 1:** Distribution of the top 10 elements in each data source.

Code and verbatim description				Record, n (%)
**Genes**				
	*TP53*	tumor protein p53	553 (54.70)	
	*KRAS*	KRAS proto-oncogene, GTPase	292 (28.88)	
	*MLL2*	lysine methyltransferase 2D^a^	173 (17.11)	
	*LRP1B*	LDL receptor related protein 1B	171 (16.91)	
	*MLL3*	lysine methyltransferase 2C^a^	150 (14.84)	
	*APC*	APC regulator of WNT signaling pathway	141 (13.95)	
	*ARID1B*	AT-rich interaction domain 1B	137 (13.55)	
	*FAT1*	FAT atypical cadherin 1	134 (13.25)	
	*PRKDC*	protein kinase, DNA-activated, catalytic subunit	128 (12.66)	
	*ARID1A*	AT-rich interaction domain 1A	126 (12.46)	
**Diagnosis^b^**				
	Z02.9	Work Status Exam (RTW)	204 (25.66)	
	I10	Hypertension (HTN) Chronic	142 (17.86)	
	401.9	HYPERTENSION NOS	138 (17.36)	
	272.4	HYPERLIPIDEMIA NEC/NOS	116 (14.59)	
	R91.8	Mass Lung	113 (14.21)	
	V68.9	ADMINISTRTVE ENCOUNT NOS	106 (13.33)	
	Z00.00	Maintenance Health (HM)	101 (12.70)	
	E78.5	Dyslipidemia NOS	93 (11.70)	
	V72.83	PREOP EXAMINATION NEC	79 (9.94)	
	V70.0	ROUTINE MEDICAL EXAM	79 (9.94)	
**Lab tests^c^**				
	777-3	Platelets [#/volume] in Blood by Automated count	991 (99.40)	
	2160-0	Creatinine [Mass/volume] in Serum or Plasma	988 (99.10)	
	965763	Hematocrit [Volume Fraction] of Blood by Automated count	985 (98.80)	
	718-7	Hemoglobin [Mass/volume] in Blood	985 (98.80)	
	788-0	Erythrocyte distribution width [Ratio] by Automated count	985 (98.80)	
	789-8	Erythrocytes [#/volume] in Blood by Automated count	985 (98.80)	
	1749545	Leukocytes [#/volume] in Blood by Automated count	985 (98.80)	
	787-2	MCV [Entitic volume] by Automated count	985 (98.80)	
	337180	Potassium [Moles/volume] in Serum or Plasma	975 (97.80)	
	383903	Sodium [Moles/volume] in Serum or Plasma	973 (97.60)	
**Family historical records^b^**				
	V47.2	Other cardiorespiratory problems	205 (29.54)	
	429.9	Heart disease, unspecified	205 (29.54)	
	429.89	Other ill-defined heart diseases	205 (29.54)	
	162.9	Malignant neoplasm of bronchus and lung, unspecified	133 (19.16)	
	162.8	Malignant neoplasm of other parts of bronchus or lung	130 (18.73)	
	272.4	Other and unspecified hyperlipidemia	124 (17.87)	
	434.91	Cerebral artery occlusion, unspecified with cerebral infarction	104 (14.99)	
	799.9	Other unknown and unspecified cause of morbidity and mortality	84 (12.10)	
	311	Depressive disorder, not elsewhere classified	72 (10.37)	
	447.9	Unspecified disorders of arteries and arterioles	63 (9.08)	
**Medication^d^**				
	5956	Iohexol	399 (72.41)	
	1359867	Sodium Chloride 9 MG/ML Prefilled Syringe	374 (67.88)	
	1807638	20 ML Sodium Chloride 9 MG/ML Injection	304 (55.17)	
	1807639	1000 ML Sodium Chloride 9 MG/ML Injection	298 (54.08)	
	1740467	2 ML Ondansetron 2 MG/ML Injection	251 (45.55)	
	4337	Fentanyl	224 (40.65)	
	314659	heparin sodium, porcine	207 (37.57)	
	847630	Calcium Chloride 0.0014 MEQ/ML / Potassium Chloride 0.004 MEQ/ML / Sodium Chloride 0.103 MEQ/ML / Sodium Lactate 0.028 MEQ/ML Injectable Solution	202 (36.66)	
	198440	Acetaminophen 500 MG Oral Tablet	188 (34.12)	
	1808234	10 ML Propofol 10 MG/ML Injection	163 (29.58)	
**Cancers^b^**				
	162.9	Malignant neoplasm of bronchus and lung, unspecified	231 (22.85)	
	153.9	Malignant neoplasm of colon, unspecified site	124 (12.27)	
	155	Malignant neoplasm of liver, primary	118 (12.67)	
	157.9	Malignant neoplasm of pancreas, part unspecified	116 (11.47)	
	183	Malignant neoplasm of ovary	85 (8.41)	
	185	Malignant neoplasm of prostate	80 (7.91)	
	171.9	Malignant neoplasm of connective and other soft tissue, site unspecified	68 (6.73)	
	193	Malignant neoplasm of thyroid gland	55 (5.44)	
	174.9	Malignant neoplasm of breast (female), unspecified	53 (5.24)	
	—^e^	—	—	

^a^Current standard gene symbols: *MLL2* is now *KMT2D*; *MLL3* is now *KMT2C.*

^b^International Statistical Classification of Diseases (ninth revision) code and description.

^c^LOINC code and description.

^d^RxNorm code and description.

^e^A tenth item is not included.

### Data Preprocessing and Data Modeling Based on FHIR and RDF

**Figure 2:**
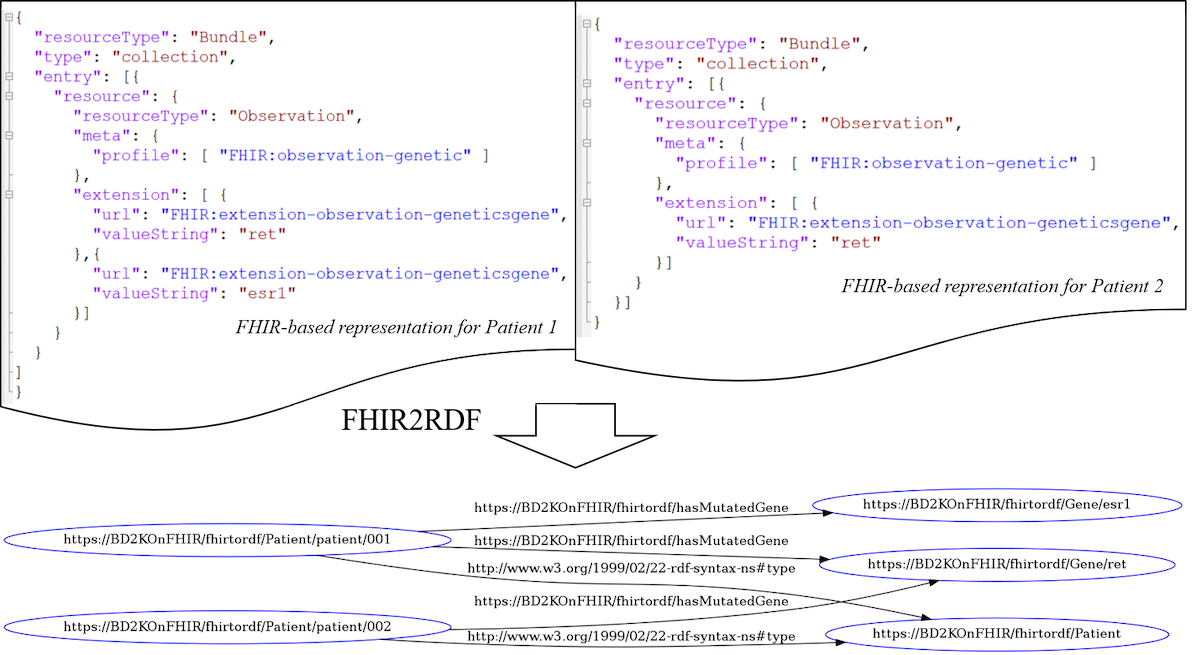
An example of data representation based on Fast Healthcare Interoperability Resources (FHIR) and Resource Description Framework (RDF): 2 JavaScript object notation–formatted FHIR representations for patients 1 and 2 are merged and converted into 1 RDF graph.

We adapted FHIR-based data models from our previous work [37] employing FHIR resources to represent data elements of genetic reports and structured electronic health record data for phenome-wide association studies. Specifically, we represented *genetic* entries with the existing profile *Observation-genetics*, extended from the resource *Observation*. The *lab test*, *diagnosis*, and *medication* entries were represented with the resources *Observation*, *Condition*, and *Medication*, respectively, and were identified by encounters (eg, billing and electronic health record encounters) and service date. The *family historical records* entities were represented with the resource *FamilyMemberHistory* as diseases and were encoded with the attributed condition. All the resources were associated with the resource *Patient*. We further converted the JavaScript object notation–formatted FHIR data to RDF format based on the conversion rules, where (1) all the string-type values were considered as the entities in the RDF graph, and (2) all the values of the resources were considered as the object of the data-type property—named after the resource for the subject resource *Patient*. We illustrated an example of data representation based on FHIR and RDF in Figure 2.

### Feature Generation and Cancer Prediction

#### Bag of Features

Bag of features is analogous to the bag-of-words representation and characterizes a sample with an orderless collection of features [38]. In this study, we used bag of features based on the attribute values from the FHIR model. Specifically, categorical values of mutated genes, lab test results, disease diagnoses, medications for treatment, and historical family disease diagnoses were collected as the features from *Observation-genetics*, *Observation*, *Condition*, *Medication*, and *FamilyMemberHistory*, respectively. Additionally, patient demographic features, such as age and gender, were also used.

#### Topological Features

In order to train a model with the features generated from the input RDF data, we adapted a methodology [21] that considered RDF graph as a network, *G*(*V*,*E*) with a set of vertices *V* and a set of edges *E*, where *V* has 7 types of vertices (ie, genetics, lab tests, diagnosis, medication, family historical records, demographics, and patients) and *E* represents associations between the 6 types of vertices (ie, genetics, lab tests, diagnosis, medication, family historical records, demographics) and patients. We used the graph embedding method to learn the features of the patients, where a patient could be represented by a vector embedded within the topological structure of the patient in the network *G*. Node2vec [30] is a state-of-art graph embedding method that vectorizes the vertices of a network based on the topology of the network by maximizing the probability of observing the neighborhood *N*(*u*) of each node *u* in *G*:

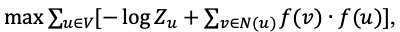



where

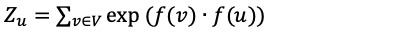



and *f* (∙) was the feature representation of a node. In addition, we also generated a |*V*|×|*V*| adjacency matrix from *G*, where each cell of the matrix was set to 1 if there was a connection between nodes, otherwise the cell was set to 0.

We modeled cancer prediction as a multiple-label classification problem, where a given patient was represented with k-dimensional features, and a model categorized the patient into precisely 1 of 9 cancer types: colon cancer (ICD-9: 153.9), pancreas cancer (ICD-9: 157.9), ovary cancer (ICD-9: 183), prostate cancer (ICD-9: 185), connective and other soft tissue cancer (ICD-9: 171.9), thyroid gland cancer (ICD-9: 193), breast cancer (ICD-9: 174.9), liver cancer (ICD-9: 155), and bronchus and lung cancer (ICD-9: 162.9).

### Experiment Design

#### Overview

There were 2 main drivers of this study: (1) from a methodological perspective—how could generated features be coordinated with classification methods in a favorable manner to achieve satisfactory prediction?—and (2) from a data perspective—which data sources, especially genetic data, are preferable in prediction? Our experiment was thus conducted as a sequence of 6 distinct tasks.

#### Task 1: Comparison of Combinations of Features and Popular Classification Methods

A comparison of 3 feature generation methods—bag of features, Node2vec, and bag of features+Node2vec (ie, a linear combination of bag of features and Node2vec)—was conducted. Seven classification methods—random forest [39], naive Bayes [40], logistic regression [41], support vector machine [42], deep neural network [43], convolutional neural network [44], and graph convolutional networks [45]—were used.

#### Task 2: Comparison of Combinations of Data Sources

There were 5 types of data sources used in this study. We took all possible combinations of the data sources into consideration and studied how the features generated from these sources affected the results.

#### Task 3: Comparison of Predictions for Each Cancer

To understand how the prediction varied in different cancers predictions, we conducted 9 prediction tasks for all the cancers to study.

#### Task 4: Analysis of Feature Contribution for Each Cancer Prediction

To interpret the model and understand which features were important to each cancer, we studied the features that contributed most to the prediction of cancer.

#### Task 5: Time Effect of Cancer Prediction

To understand how the prediction could be made precisely prior to a certain amount of time of the diagnosis, we studied the prediction based on data collected at different duration, ranging from 0 to 24 months, in advance.

#### Task 6: Prediction of Cancer of Unknown Primary Patients

We identified the 43 primary cancers from 81 patients with cancer of unknown primary based on the diagnosis records to understand how the proposed method performed for real cancer predictions. Please note, no patients with pancreas cancer of unknown primary were identified, and therefore, pancreatic cancer was not considered in this task.

### Feature Selection and Classification

Two methods were used to generate features: bag of features and Node2vec. For bag of features, all genes, diseases, drugs in genetics, diagnosis, medication, and family historical records were considered as features. For the lab tests*,* the values were converted into categorical values (Null, Normal, or Abnormal) based on the normal range defined in the unified data platform. To avoid overfitting, the features were reduced to *d*={10,20,30,40,50,60,70,80,90,100} based on information gain [46]. For Node2vec, the parameter ranges for the grid search were specified as the number of walks γ={10,40}, return *P*={0.5,1.0,2.0}, in-out *q*={0.5,1.0,2.0}, dimension *d*={10,20,30,40,50,60,70,80,90,100}, window size *w*={5,10}, and walk length *t*={40,80}.

Four popular machine learning models and 3 deep learning models were used for classification. For machine learning methods, the following settings were used: L2 regularization for logistic regression, type C-SVC and linear kernel for support vector machine, 500 trees for random forest, and default settings for naive Bayes. For deep learning methods, the following structure were used: 5 dense layers with dimensions {256, 256,128, 64, 10} (4 rectified linear unit [ReLU] activation functions with 0.5 dropout rate and 1 softmax activation function) for deep neural network, 3 convolution layers with filters {256, 256, 256} (3 ReLU activation functions and maxpooling layers with 0.5 dropout rate) followed with 4 dense layers with dimensions {256,128, 64, 10} (3 ReLU activation functions with 0.5 dropout rate and 1 softmax activation function) for convolutional neural network, and 2 graph convolutional layers with channels {64, 10}(1 ReLU activation function with 0.5 dropout rate and 1 softmax activation function) for graph convolutional networks.

Node2vec was obtained from the Node2vec library [47]. The logistic regression classifier was obtained from the LIBLINEAR library [48]; naive Bayes, random forest, and information gain algorithms were obtained from Weka library [49], support vector machine was obtained from LIBSVM [50]. Deep neural network and graph convolutional networks were constructed based on Keras library [51]. Graph convolutional networks algorithms were obtained from Spektral library [52].

### Validation and Evaluation Metrics

We used conventional 10-fold cross-validation for the evaluation, where 10 independent iterations of training and testing were conducted, and a random partition of the original samples into 10 equal-size subsamples was performed. To assess the quality of classification, we used area under the receiver operating characteristic curve (AUROC) [53]. In addition, the area under the precision-recall curve (AUPRC) [53] was used as a supplementary metric characterizing the results for imbalanced classes [54,55]. AUROC and AUPRC scores were calculated using the Java Receiver Operating Characteristic library [56] and Weka evaluation package [57].

## Results

### Combinations of Features and Popular Classification Methods

Table 2 shows the best performance result was achieved by using bag of features+Node2vec and random forest (AUROC 96.19%) (AUPRC: Table S1, Multimedia Appendix 1). Generally, using bag of features+Node2vec outperformed using bag of features (+1.27 %) and Node2vec (+1.41%). Although we observed that machine learning–based methods outperformed deep learning–based methods, in general, the best deep learning–based approach (AUROC 95.12%) was second to the best machine learning–based approach by only 1 percentage-point difference (outperforming the remaining machine learning–based approaches). As our implementation of deep learning models is based on simple architectures, the deep learning models with more complex architectures have the potential to facilitate feature generation and may directly contribute to improvements in cancer prediction.

**Table 2:** Prediction performance (area under the receiver operatic characteristic curve) for combinations of features and classification methods.

Classifiers	Feature generation algorithm		
	Bag of features	Node2vec	Bag of features+Node2vec
	AUROC^a^ (%)	AUROC (%)	AUROC (%)
Random forest	94.82	91.89	96.19
Naive Bayes	92.30	92.91	94.76
Logistic regression	86.68	85.25	89.39
Support vector machine	84.62	83.92	86.72
Convolutional neural network	64.14	63.36	57.68
Deep neural network	92.56	92.87	95.12
Graph convolutional networks	79.67	83.62	83.83

^a^AUROC: area under the receiver operating characteristic curve.

### Combinations of Data Sources

Table 3 shows better results were achieved by the model DML+G (diagnosis, medication, lab test, and genetic information; AUROC 96.56%). Steady improvement is obtained when more features are used (AUPRC: Table S2, Multimedia Appendix 1). For example, increasing average AUROCs (75.49%, 82.65%, 87.98%, and 91.74%) are achieved by adding 1 to 5 features successively without using genetic information. Table 3 also presents the importance of the features, where lab test is the most important feature (91.00%), followed by diagnosis (73.12%), medication (72.83%), and family historical records (65.01%). We also demonstrated the value of genetic information for cancer prediction—an average improvement of 10.52% was reached. Interestingly, such improvement is weakened when more feature types are used (+15.76% for using 1 feature type, +10.45% for 2 feature types, +6.92% for 3 feature types, and +4.45% for 4 feature types). Table 3 also indicates the potential of using diverse types of features alternatively when genetic information is not available.

**Table 3:** Prediction performance for combinations of data sourcing with bag of features+Node2vec and random forest algorithms.

Feature types		AUROC^a^ (%)			
			Base feature set		With genetic information
**1 feature type**					
	G^b^		73.12		90.89
	D^c^		65.01		88.37
	H^d^		91.00		95.80
	L^e^		72.83		89.94
	M^f^		73.21		90.92
**2 feature types**					
	DH		91.55		96.09
	DL		77.09		90.88
	DM		91.30		95.92
	HL		71.53		89.02
	MH		91.22		95.75
	ML		91.98		96.01
**3 feature types**					
	DHL		76.76		91.28
	DMH		91.76		96.56
	DML		91.43		95.76
	MHL		91.74		96.19
**4 feature types**					
	DMHL		73.12		90.89

^a^AUROC: area under the receiver operating characteristic curve.

^b^G: genetic information.

^c^D: diagnosis.

^d^H: family historical records.

^e^L: lab test.

^f^M: medication.

### Predictions for Each Cancer

Table 4 shows that the proposed method achieved high AUROC values across all 9 cancer types (AUPRC: Table S3, Multimedia Appendix 1), especially for thyroid gland (AUROC 99.80%), prostate (99.76%), breast (98.53%), ovary (98.29%), connective and other soft tissue (96.05%), and liver (95.41%). Genetic information improved the predictions in general (*P*<.001) based on a Wilcoxon signed-rank test [58], specifically for thyroid gland cancer (*P*=.03), ovary cancer (*P*=.03), connective and other soft tissue cancer (*P*=.03), liver cancer (*P*=.03), and colon cancer (*P*=.03).

**Table 4:** Prediction performance for 9 cancer types.

Cancer (ICD-9^a^ code)	AUROC^b^ (%)	
	DML^c^	DML+G^d^
Malignant neoplasm of thyroid gland (193)	99.55	99.80
Malignant neoplasm of prostate (185)	98.43	99.76
Malignant neoplasm of breast (female), unspecified (174.9)	96.80	98.53
Malignant neoplasm of ovary (183)	95.73	98.29
Malignant neoplasm of connective and other soft tissue, site unspecified (171.9)	82.39	96.05
Malignant neoplasm of liver, primary (155)	91.39	95.41
Malignant neoplasm of pancreas, part unspecified (157.9)	91.07	95.41
Malignant neoplasm of bronchus and lung, unspecified (162.9)	90.61	93.24
Malignant neoplasm of colon, unspecified site (153.9)	79.88	92.56

^a^ICD-9: International Statistical Classification of Diseases, ninth revision.

^b^AUROC: area under the receiver operating characteristic curve.

^c^DML: diagnosis, medication, and lab test.

^d^DML+G: diagnosis, medication, and lab test, and genetic information.

### Feature Contributions for Each Cancer Prediction

**Figure 3:**
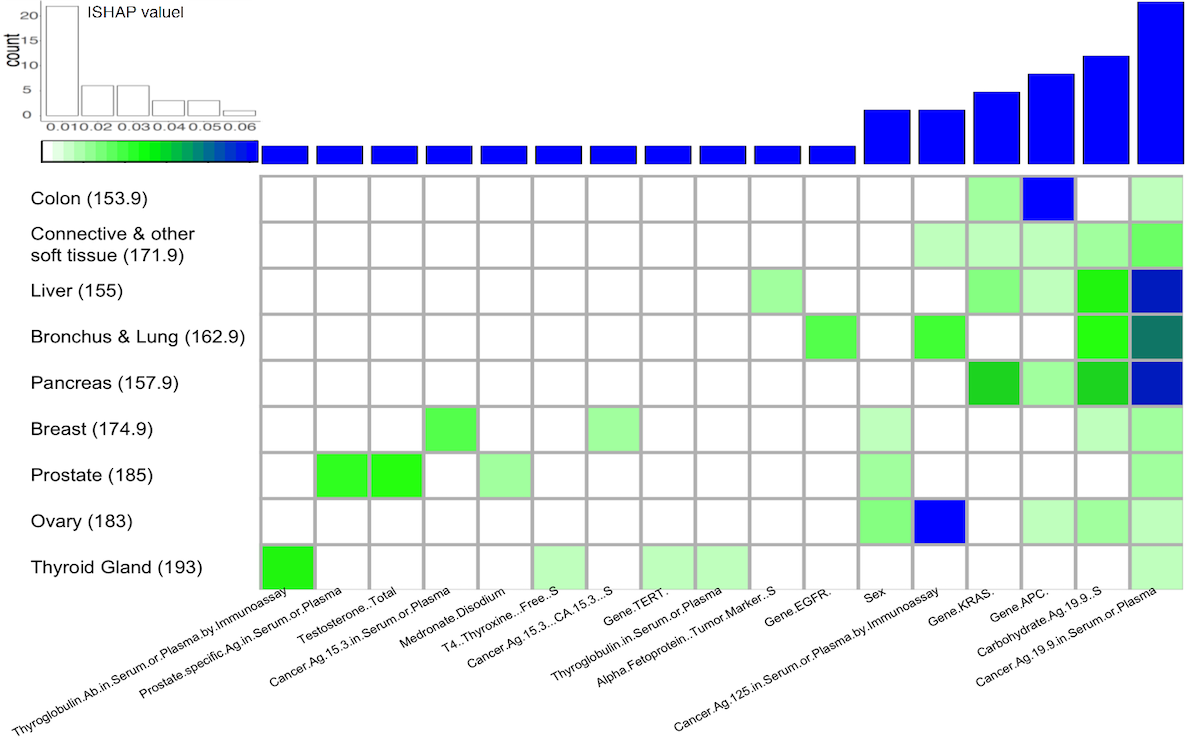
Top 5 features contributing to cancer prediction.

Our analysis examines the feature contribution based on SHAP values [59] for the cancer prediction and selects the top 5 features interpretable for each cancer (Figure 3). Frequent common features are lab tests (11/17); cancer antigen 19-9 in serum or plasma (2.03%), carbohydrate antigen 19-9, S (1.76%), and cancer antigen 125 in serum or plasma by immunoassay (2.59%) are the most common features across all the cancer types. These lab tests are considered to be predictive biomarkers for prognosis and chemotherapeutic effect for carcinomas [60-63]. Two genes—KRAS proto-oncogene, GTPase homolog (*KRAS*) (1.46%) and adenoma polyposis coli regulator of WNT signaling pathway (*APC*) (1.60%) contribute the most cancer predictions. *KRAS* is the most commonly mutated oncogene in human cancers. The sustained expression and signaling of *KRAS* results in the progress of many cancers thus make it the high-priority target in clinical therapeutic implications [64]. *APC* participates in a cytoplasmic complex and its mutation triggers negatively regulating canonical WNT signaling. *APC* counteracts proliferation, facilitates apoptosis, and suppresses tumor progression, thus APC-deficient tumors drive colorectal and gastric cancers [65,66].

Lab tests testosterone (2.49%) and prostate-specific antigen in serum or plasma (2.29%) were found to be the major contributors to prostate cancer prediction. Evidence supports the androgen hypothesis, where prostate cancer development and progression are related to androgens. These findings drive the studies to explore the correlation between testosterone and prostate cancer development and progression [67,68]. For thyroid gland cancer prediction, thyroglobulin antibody in serum or plasma by immunoassay (2.69%), thyroglobulin in serum or plasma (0.58%), T4 (thyroxine) (0.62%), and gene telomerase reverse transcriptase (*TERT*) (SHAP value 0.59%) were found to be the major contributors. Associations between autoimmune thyroiditis and thyroid cancer have been documented [69] in studies where thyroid autoimmunity was assessed by measuring thyroglobulin antibody and thyroid peroxidase antibody [70,71]. Thyroglobulin in serum also plays a key role in the surveillance of differentiated patients with thyroid cancer [72]. *TERT* promoter mutations have been found to be strongly associated with different pathological types of thyroid cancers and are considered as the biomarker to the preoperative diagnosis and prognosis of thyroid cancers [73]. Cancer antigen 15-3 in serum or plasma (1.57%) and cancer antigen 15-3 (CA 15-3) S (0.98%) lab tests are the major contributors to breast cancer prediction. Cancer antigen 15-3 is a protein made by a variety of cells, particularly breast cancer cells, and the cancer antigen 15-3 test is A biomarker test used to monitor breast cancer [74]. In addition, the cancer markers alpha-fetoprotein, tumor marker, S (0.78%) and epidermal growth factor receptor (EGFR) (1.56) were found to be the main contributors for the prediction of cancers of the liver [75] and bronchus and lung [76]. In our study, sex appears to be the major contributor to prediction of cancers of the breast (0.76%), prostate (0.92%), and ovary (1.16%).

### Time Effect of Cancer Prediction

Table 5 shows predictions based on different resources with different combinations of time-dependent (diagnosis, medication, and lab test) features (AUPRC: Table S4, Multimedia Appendix 1). Among the 7 models, diagnosis and lab test were the best (average AUROC 90.31 %). In general, the performance of prediction decreases as more time increases prior to the formal diagnosis. For example, the average performance was reduced from 92.37% to 77.18% from 0 months to 24 months in advance, with an average decrease of 3.04%. Table 5 also demonstrates the performance of the model (ie, diagnosis, medication, lab test, and genetic information) based on genetic information (AUROC 91.38% at 24 months in advance, an improvement of +11.38% over diagnosis, medication, and lab test). The difference between the two increase as time increases (eg, 1.06 for 0 months to 11.38% for 24 months), which suggests the importance of genetic testing at early stages.

**Table 5:** Prediction performance (AUROC) 0 months to 24 months in advance.

Months	Feature type							
	DML+G^a^	Diagnosis, medication, and lab test	Diagnosis and lab test	Diagnosis and medication	Medication and lab test	Diagnosis	Medication	Lab test
	AUROC^b^ (%)	AUROC (%)	AUROC (%)	AUROC (%)	AUROC (%)	AUROC (%)	AUROC (%)	AUROC (%)
0	99.43	98.36	98.41	97.89	88.67	97.90	70.39	87.93
1	98.08	95.62	95.51	94.31	86.83	94.53	71.01	86.67
3	96.52	93.16	93.22	90.74	84.85	91.20	69.36	84.18
6	95.21	89.69	89.91	85.53	83.09	85.26	68.12	83.38
12	93.17	84.39	84.60	78.20	80.56	78.21	66.76	79.99
24	91.38	80.01	80.20	71.81	77.73	71.71	66.22	78.35

^a^DML+G: diagnosis, medication, and lab test, and genetic information.

^b^AUROC: area under the receiver operating characteristic curve.

### Prediction of Patients With Cancer of Unknown Primary

In spite of the challenge in identifying patients with cancer of unknown primary in the clinical setting, hybrid features—the diagnosis, medication, lab test, and genetic information model—outperformed the diagnosis, medication, and lab test model (AUPRC: Table S5, Multimedia Appendix 1), and bag of features+Node2vec outperform the bag of features and Node2vec in most cases. Table 6 shows promising prediction results for 4 cancers, especially for breast (AUROC 92.31%), connective and other soft tissue (AUROC 92.31%). Cancers of the liver and lung have the largest number of patients (24/43) and also achieved satisfactory predictions (AUROCs 88.21% and 85.51%). We also note that the proposed method performed suboptimally in predicting cancer of the colon (AUROC 52.56%). Prediction of the prostate, thyroid gland, and colon cancers had better results for the bag of features+Node2vec model with diagnosis, medication, and lab test features and for the bag of features or Node2vec model with diagnosis, medication, lab test, and genetic information features (Table S6, Multimedia Appendix 1), suggesting a more flexible strategy of model adaptation for the prediction of cancer of unknown primary in practice.

**Table 6:** AUROC (%) of prediction for 9 cancer types.

Cancer (ICD-9^a^ code)	AUROC^b^ (%)			Patients, n
	DML^c^	DML+G^d^		
Malignant neoplasm of breast (female), unspecified (174.9)	83.97	92.31	4	
Malignant neoplasm of connective and other soft tissue, site unspecified (171.9)	53.21	92.31	4	
Malignant neoplasm of liver, primary (155)	84.10	88.21	13	
Malignant neoplasm of bronchus and lung, unspecified (162.9)	74.43	85.51	11	
Malignant neoplasm of ovary (183)	65.85	80.49	2	
Malignant neoplasm of prostate (185)	91.67	79.17	3	
Malignant neoplasm of thyroid gland (193)	90.24	75.61	2	
Malignant neoplasm of colon, unspecified site (153.9)	64.74	52.56	4	

^a^ICD-9: International Statistical Classification of Diseases, ninth revision.

^b^AUROC: area under the receiver operating characteristic curve.

^c^DML: diagnosis, medication, and lab test.

^d^DML+G: diagnosis, medication, and lab test, and genetic information.

## Discussion

It is recognized that both genetic and nongenetic factors may lead to the development of cancers, and they are, therefore, considered to be risk factors in the plethora of cancer prediction models based on statistical analysis; this leads to performance (eg, AUROC) ranging from 60% to 90% [77]. For example, the variables of high DNA load of high-risk human papillomavirus, age, marital status, smoking status, and age at sexual debut are the critical factors to achieve the AUROC 90% in the prediction of cervical intraepithelial neoplasia grade 2 or worse [78]. DNA methylation-based markers-based method achieves AUROC 93% in the detection of preinvasive neoplasia and cervical cancer [79]. Computational methods (eg, machine learning and deep learning) have been adapted to provide solutions for cancer prediction challenges in a controlled environment (eg, UCI machine repository [80]). For example, linear support vector machines achieved AUROC 96.7% [81] and k-nearest neighbors classifier achieved an accuracy of 99.28% [82] for breast cancer prediction.

Public genetic expression databases (eg, The Cancer Genome Atlas) are frequently used to train diverse deep learning models. A convolutional neural network–based model achieved accuracies of 93.9% to 95.0% in the prediction of 34 cancer types [83]. For lung, stomach, and breast cancer, AUROCs 99.5%, 97.1%, and 95.0%, respectively, were achieved by a stacked sparse auto-encoder–based classification model [84]. Prostate cancer prediction achieved an AUROC of 95.5% with a genetic algorithm–optimized artificial neural network [85]. Accuracies of 95.3% for breast cancer, 57.9% for leukemia, and 84.9% for colon cancer were achieved by sample expansion based 1D convolutional neural network [86].

Electronic health records are utilized in cancer prediction. DeepPatient has proposed a novel unsupervised feature learning method based on autoencoders for disease prediction [87]. The overall AUROC was 77.3%, where AUROCs of 88.7% for cancer of rectum and anus, 88.6% for cancer of the liver and intrahepatic bile duct, 85.9% for cancer of the prostate were predicted with a time interval of 12 months. Multiple studies have utilized electronic health record data to predict specific cancers, where AUROCs of 88.1% for lung cancer [88], 64.8% for breast cancer [89], 85% for pancreatic cancer [90] were achieved, and 85.7% precision and 60.0% recall were achieved for colorectal cancer [91]. Our method achieved AUROC 96.56% in general and outperformed the state-of-the-art methods for most cancer types. Specifically, prostate cancer (99.8%), breast cancer (AUROC 98.5%), liver cancer (95.4%), and pancreas cancer (95.4%) predictions results were better for our method.

In this study, we designed and developed a network-based framework leveraging the FHIR resources and RDF for cancer prediction. Our contributions can be summarized as exploration of utilizing FHIR and RDF technology to provide a network-based representation for the prediction of patient health status, demonstrating the value of integrating the phenotypic and genetic features data sources to improve the accuracy and interpretability in cancer prediction models. To enable the standard representation of data, a FHIR-based representation was used as the core to support the network population and feature generation. It is one of the most popular clinical data standards and is widely used among modern electronic health record vendors and data providers, enabling the plug and play functionality of the proposed method to be used across the different institutions, and it provides the specification and tools to seamlessly convert to RDF format and support the efficient data communication based on the popular data exchanging formats, such as XML or JavaScript object notation.

This study demonstrated a solution for the prediction of unknown cancer in clinical practice. Despite the value of this work, there are several limitations that should be addressed.

First, the genetic alterations in the genetic reports provided in Foundation Medicine are all somatic mutations in tumors and are collected from somatic tissues. Thus, we could not differentiate the germline and somatic mutations in our model. The bias introduced to the system caused by a failure in capturing this difference weakens the findings of our study.

Second, as most genetic tests are based on specimens collected from the biopsy or surgery, the best-performing (diagnosis, medication, lab test, and genetic information) model introduced in Task 5 might not be adaptable as some medical organizations have limited access to genetic information available for study. We, therefore, consider that it is more practical to learn a large amount of phenotypical information for cancer prediction with the full utilization of existing generic information. On the other hand, as the costs of genetic testing are reduced, we believe that the genetic information will be increasingly used in prediction models for different tasks, which makes the proposed method a good reference as a pilot study.

Third, within 81 patients who have been documented as having cancer of unknown primary (from genetic reports), we could identify specific cancer types for 43 patients based on the review of patients' diagnostic report for task 6. We understand that the limited data set used might affect result analysis, which is a limitation of this experiment. We also noticed that the proposed method performs differently in task 6, especially with some notable failures. Such failures indicate the patterns of the value distribution for the features learned in the training data are not the same as the patterns in the cancer of unknown primary. The cancer of unknown primary source is not considered a single type of cancer and is known to spread at the early stage without causing phenotypical symptoms at the origin site [92]. As such, the proposed model is affected in Task 5 accordingly.

Fourth, our experiment demonstrates the performance of the proposed method based on data collected over a varying timeline. Data were used in isolation to train classification models, ignoring the continuous changing of the measurable values of phenotypes (eg, lab tests) during cancer progression. The introduction of deep learning models, such as recurring neural networks [93] and long short-term memory [94], which are capable of processing time-series data may potentially improve predictions.

Fifth, cancers related to the same genetic alteration (eg, both colorectal and gastric cancers are related to the *APC* gene) inspire us to explore the potential of considering dependent phenotypes of the genetic alteration. With the utilization of phenotype and genotype dependence based on the ontology structure, a more sophisticated method can be designed to empower the prediction. In the future, we plan to reach out to other institutions to apply our method both with and without genetic information on diverse electronic health record systems. We consider it is necessary to adopt other medical data standards, such as Observational Health Data Sciences and Informatics Common Data Model [95], to cover the diversity. We are aware that there are some challenging issues in genetic data modeling with relational databases, such as how to anonymize and aggregate genomic data. We believe that the research community will develop solutions for handling these challenging issues. We will incorporate such developments into our framework as part of future work to better support these requirements. The data process and cancer prediction tools of this study are publicly available [96].

## Appendix

Multimedia Appendix 1 Supplementary tables.

## Abbreviations

AUPRC: area under the precision–recall curve
AUROC: area under the receiver operating characteristic curve
ICD: International Statisitical Classification of Diseases
FHIR: Fast Healthcare Interoperability Resources
RDF: Resource Description Framework
ReLU: rectified linear unit
